# Early exhumation of the Frontal Cordillera (Southern Central Andes) and implications for Andean mountain-building at ~33.5°S

**DOI:** 10.1038/s41598-019-44320-1

**Published:** 2019-05-28

**Authors:** Magali Riesner, Martine Simoes, Daniel Carrizo, Robin Lacassin

**Affiliations:** 1 Université de Paris, Institut de physique du globe de Paris, CNRS, F-75005 Paris, France; 2 now at CEA, DAM, DIF, F-91297 Arpajon, France; 3 Advanced Mining Technology Center, University de Chile, Santiago, Chile

**Keywords:** Geology, Tectonics

## Abstract

The Andes are the modern active example of a Cordilleran-type orogen, with mountain-building and crustal thickening within the upper plate of a subduction zone. Despite numerous studies of this emblematic mountain range, several primary traits of this orogeny remain unresolved or poorly documented. The onset of uplift and deformation of the Frontal Cordillera basement culmination of the Southern Central Andes is such an example, even though this structural unit appears as a first-order topographic and geological feature. To solve for this, new (U-Th)/He ages on apatite and zircon from granitoids of the Frontal Cordillera at ~33.5°S are provided here. These data, interpreted as an age-elevation thermochronological profile, imply continuous exhumation initiating well before ~12–14 Ma, and at most by ~22 Ma when considering the youngest zircon grain from the lowermost sample. The age of exhumation onset is then refined to ~20 Ma by combining these results with data on sedimentary provenance from the nearby basins. Such continuous exhumation since ~20 Ma needs to have been sustained by tectonic uplift on an underlying crustal-scale thrust ramp. Such early exhumation and associated uplift of the Frontal Cordillera invalidate the classically proposed east-vergent models of the Andes at this latitude. Additionally, they provide further support to recent views on Andean mountain-building proposing that the Andes at ~33.5°S grew firstly over west-vergent basement structures.

## Introduction

**Figure 1:**
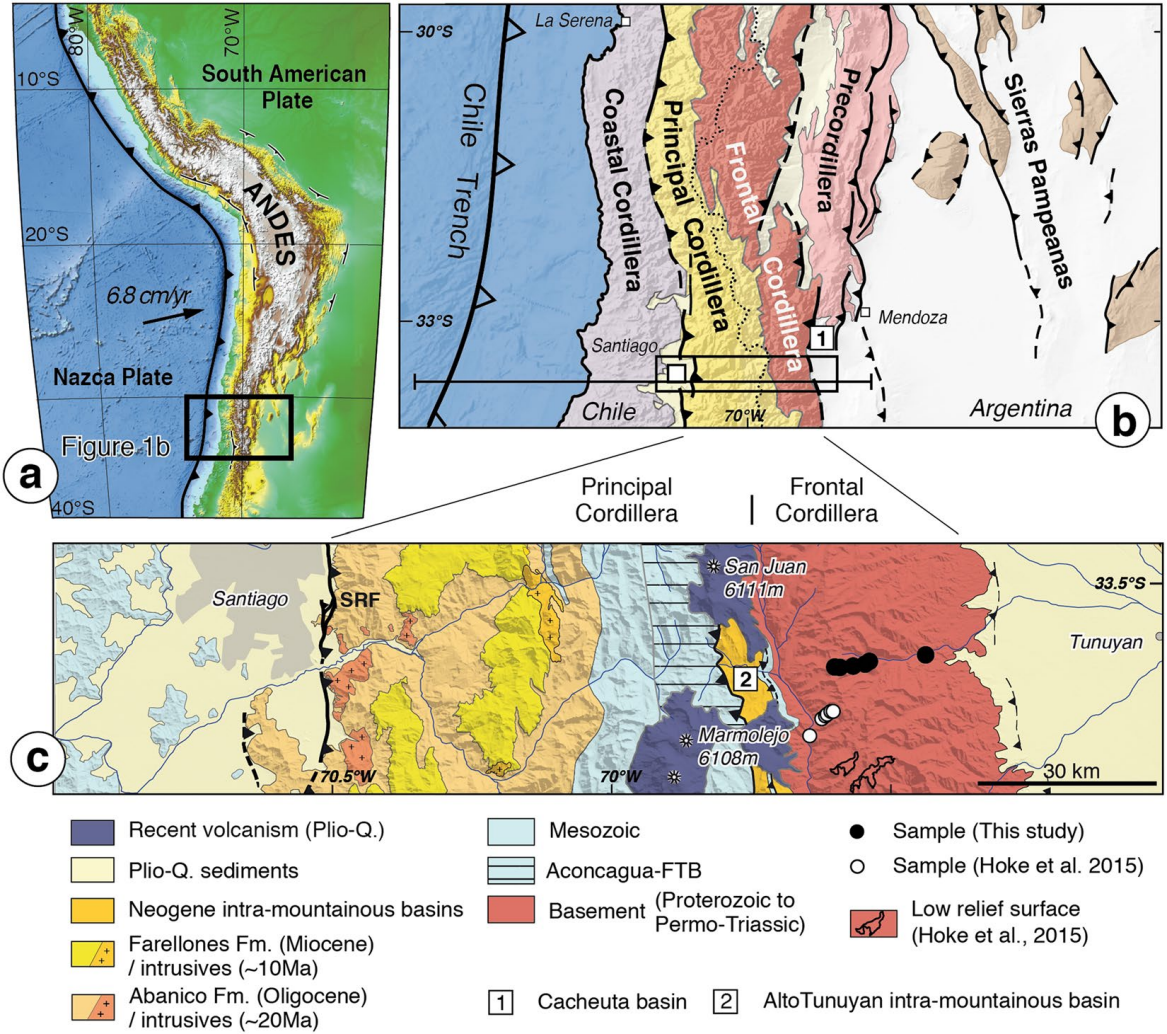
Location and geological background of the study area. (**a**) Topography of the Andes, along the boundary between South America and the subducting Nazca Plate. Box locates (**b**). (**b**) Structural scheme of the Southern Central Andean subduction margin, picturing the principal structural units. At ~33.5°S the Andes are structurally relatively simple and are constituted of the Principal and Frontal Cordilleras. The crustal-scale cross-sections of Fig. 5 are located. Box locates (**c**). (**c**) Structural scheme of the study region compiled from geological maps (^23^ and references therein), reporting the location of existing thermochronological ages within the Frontal Cordillera basement with black (this study) and white dots^43^. Black contours on the basement represent low relief surfaces as mapped by Hoke *et al*.^43^. FTB: fold-and-thrust belt; SRF: San Ramón Fault; (1): Cacheuta Basin (in **b**); (2): Alto Tunuyan intra-mountainous basin (in **c**). Digital Elevation model is extracted from SRTM3 data. Figure was generated with Adobe illustrator CS6 (http://www.adobe.com/fr/products/illustrator.html).

The Andes extend over ~4500 km along the western margin of the South America continental plate (Fig. 1a). This mountain belt stands as one of the highest topographic regions on Earth and is the only present-day active example of a Cordilleran- (or subduction-) type orogen, i.e. of mountain-building within the upper plate of a subduction zone^1^. Despite the long-lived subduction along the western margin of South America since the Early Mesozoic, Andean mountain-building only occurred since the Late Cretaceous - Early Cenozoic. Initiation of mountain-building has been proposed to be related to the existence of flat-slab segments (e.g.^2,3^), to the evolving age of the subducting slab^4^, to the large dimensions of the subduction zone^5^, to the westward drift of South America^6^ or to the penetration of the slab into the lower mantle^7^. The Andes are characterized by significant lateral variations in width (Fig. 1a) and cumulative shortening (e.g.^8,9^), which have been interpreted as either related to the structural inheritance of a laterally segmented South American foreland (e.g.^10^), to varying boundary conditions along the subduction zone (e.g.^8,11,12^), to variable rates and timing of deformation (e.g.^13^), or a combination of these factors. The first-order kinematics as well as mechanics of Andean mountain-building remain in fact unresolved, at the large continental scale as well as at the more regional scale of some of the best geologically documented structural sections.

Two regions have been mostly investigated and documented at the scale of the whole orogen, to the north (~18–21°S) and to the south (~33–34°S) of the Central Andes. To the north, the Andes reach their largest width of ~700 km and highest average elevations, with the presence of the emblematic Altiplano-Puna Plateau (Fig. 1a). Compressional deformation is here complex and long-lived since the Late Cretaceous - Early Cenozoic (e.g.^11,13–15^), and cumulative shortening amounts to a maximum of ~300–350 km with the progressive accretion of various structural units to the range (e.g.^13,15–18^). At ~33–34°S latitude, the Andes are narrow and structurally more simple (Fig. 1a,b), with significantly less than 100 km of cumulative shortening (e.g.^19–23^) since ~20–25 Ma (e.g.^19,22–25^). Some emblematic structures, such as the Aconcagua fold-and-thrust belt (hereafter: Aconcagua FTB), have for long attracted structural or modelling investigations (e.g.^19,21,23,26–30^). As such, this southern part of the Central Andes is expected to offer the opportunity to retrieve eventual initial stages and first-order processes of Andean mountain-building.

At ~33.5°S, the Andes are composed of fold-and-thrust belts affecting Meso-Cenozoic sedimentary and volcanic rocks of the Principal Cordillera to the west (e.g.^22–24,31^), and of the Proterozoic to Permo-Triassic pre-Andean basement of the Frontal Cordillera to the east^27,32,33^ (Fig. 1c). The regional topography, with altitudes reaching ~5 km within the Frontal Cordillera, gently tapers westward, in parallel to the overall-westward younging of the stratigraphy. Within the Principal Cordillera, the West Andean fold-and-thrust belt (hereafter: West Andean FTB) is west-vergent^34^. The active San Ramón fault^24,35^ is the most frontal ramp that connects to the basal decollement under the West Andean FTB, and reaches the surface along the western mountain front. In contrast, further east, the Aconcagua FTB is a shallow east-vergent unit thrusted over the Frontal Cordillera (e.g.^21,26,27,30^) (Fig. 1c). To the east, the Frontal Cordillera basement high is composed of Paleo-Proterozoic series affected by faults (e.g.^36^), sealed by the unconformable Permo-Triassic Choiyoi Group and with probable limited displacements after deposition of this Permo-Triassic series, in particular at ~33–33.5°S^23^. Such unconformable contact presently outlines a broad ~30–50 km wide antiformal culmination, interpreted to be related to - and illustrative of - the recent Andean deformation of the Frontal Cordillera^23^. At ~33.5°S, the amount of thrusting along the eastern front of the Frontal Cordillera is limited (e.g.^22^).

**Figure 2:**
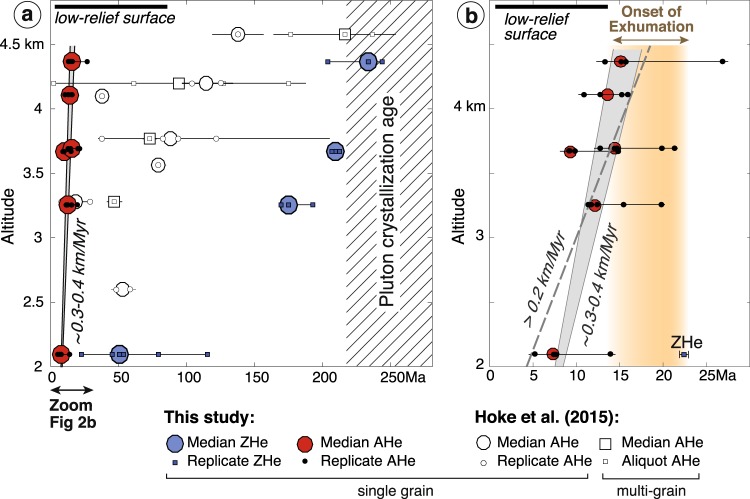
Age-elevation plots of available thermochronological data. (**a**) (U-Th)/He apatite (AHe) and zircon (ZHe) cooling ages (data from Hoke *et al*.^43^ and this study with white and colored symbols, respectively) as a function of sample altitudes. Large symbols show median ages for each sample and smaller ones report the ages of each single grain or aliquot for a given sample. The overall age dispersion within each sample is represented by horizontal lines (following Gourbet *et al*.^57^). All the data from this study are provided in detail in Tables S1 to S3 (Supplementary Materials). The pluton crystallization age is taken from published maps and data^50,51^. (**b**) Detail of the age-elevation profile for the 0–25 Ma time frame using the data obtained in this study. All AHe ages are reported. The youngest single grain ZHe age of the lowest sample is also indicated. Exhumation of the Frontal Cordillera basement was already ongoing by ~12–14 Ma (as suggested by the youngest single grain AHe age of 13.0 ± 0.7 Ma of the highest sample) and started sometime after ~22 Ma (as derived from the youngest single grain ZHe age of 22.3 ± 0.2 Ma of the lowest sample). Minimum and most probable apparent exhumation rates as inferred from our data are reported. Top of low relief surface extracted from Hoke *et al*.^43^. Figure was generated with Adobe illustrator CS6 (http://www.adobe.com/fr/products/illustrator.html).

Even though the Frontal Cordillera appears as a first-order topographic and geological feature of the Andes at ~33.5°S (Fig. 1c), comparable to other basement culminations observed in the interior of other mountain ranges worldwide (e.g.^37,38^), the timing of its Andean uplift and deformation remains poorly solved and ambiguous. At ~33.5°S, the common perception is that Andean deformation started in the Principal Cordillera and propagated from west to east, implying that the Frontal Cordillera was accreted to the mountain range and uplifted only after ~10 Ma (e.g.^20,21,27^). But most recent provenance analyses of detrital sediments from the Alto Tunuyan intramountainous basin^39^ or from the eastern Cacheuta foreland basin^40^ (Fig. 1b,c) indicate that the Frontal Cordillera has been a sedimentary source for these basins since ~16–20 Ma. In fact, the presence of conglomeratic clasts from the Frontal Cordillera early in the deposits of intramountain basins at ~33.5°S was already noticed by Darwin^41,42^ (see Supplementary Materials for further details on the observations and derived interpretation of this pioneer work). These results on sedimentary provenance have been interpreted as reflecting either an early uplift of the Frontal Cordillera or the presence of an inherited paleo-basement high that has been subsequently and only recently uplifted^39^. In addition to these sedimentary archives, recent results from (U-Th)/He thermochronology on apatite from the source rocks of the Frontal Cordillera by Hoke *et al*.^43^ (Fig. 1c) give more direct constraints on the exhumation of this basement high at ~33–33.5°S. Based on their data, these authors propose that exhumation of the Frontal Cordillera initiated early by ~25 Ma at a slow rate of ≤0.1 km/Myr. Following an indirect reasoning based on the extrapolation of their apparent exhumation rate at lower elevations and with some assumptions on the geothermal gradient, they propose that exhumation accelerated sometime after ~10 Ma, related to the onset of Andean deformation of the Frontal Cordillera. However, their single and multi-grain (U-Th)/He ages on apatite show a large degree of dispersion (Fig. 2a), making these deductions on the ages of initiation and acceleration of exhumation debatable.

This ambiguity in the timing of uplift of the Frontal Cordillera, as derived from the timing of its exhumation from the point of view of either the sedimentary source or of the sedimentary record in basins, has fed diverging tectonic models of the Andes at ~33–34°S. These models have considered either that this basement high was uplifted recently by 8–9 Ma in keeping with the idea of a sequential eastward propagation of deformation (e.g.^19,21,22,27,30^), or that its uplift has been sustained over the last ~20–25 Myr by westward slip on an underneath crustal-scale ramp^23,24^. To refine existing data on the uplift and associated exhumation of the Frontal Cordillera, new better-resolved low-temperature thermochronological ages are hereafter provided (Fig. 2). The samples were collected from plutons located in the core of this basement high in Argentina at ~33.5°S (Fig. 1c). The implications of these findings are then discussed in terms of total exhumation and of age of exhumation onset, as well as of Andean mountain-building at this latitude.

## Methodology and Sampling Strategy

Thermochronology allows for deriving the cooling of rocks on their way up to the surface, and therefore provides quantitative constraints on their exhumation history (e.g.^44,45^). Here, (U-Th)/He thermochronology on apatite (AHe) and zircon (ZHe) is used to assess the exhumation history of rocks of the Frontal Cordillera through temperatures of ~40–80 °C^46^ and ~160–200 °C^47^, respectively, typically encountered at shallow crustal depths (<10 km depth). The range of closure temperatures is referred to as the partial retention zone (hereafter PRZ) for each system. The combination of two thermochronological systems, with samples taken at different altitudes along an elevation profile, is commonly used in an age-elevation plot to retrieve the apparent exhumation rate through the underlying geothermal gradient and the evolving surface topography, and in some favorable cases the timing of onset of exhumation when total exhumation has not been sufficient to erase this record (e.g.^44,45^). Because samples are most of the time collected over a certain horizontal distance, retrieved exhumation rates are apparent, and can only be used as proxys for the actual exhumation rates (e.g.^44,48,49^). Finally, these exhumation and cooling ages are in turn often used as indirect estimates for the age and/or rate of orogenic uplift.

At ~33–33.5°S, Hoke *et al*.^43^ recently obtained AHe ages along two elevation profiles in Permo-Triassic rocks (Choiyoi Group) from the western flank of the Frontal Cordillera (Fig. 1c). The sampled lithologies were not clearly reported by the authors, but based on published regional descriptions of the Choiyoi Group^50^, it is here inferred that most of their samples correspond to volcanic (lavas, ignimbrites and tuffs) or plutonic (granitoids) rocks. Their single and multi-grain AHe ages show a large degree of dispersion of often several tens of Ma (Fig. 2a), which Hoke *et al*.^43^ interpreted as due to radiation damage during the slow cooling of the samples through the PRZ of the AHe system.

With the idea of refining Hoke *et al*.^43^’s previous results, two granite-granodiorite plutons of Permo-Triassic age (Cerro Punta Negra and Punta Blanca^50,51^) were specifically sampled within the core of the Frontal Cordillera (Argentina) at ~33.5°S (Fig. 1c). These samples come from a ~2.3 km-high elevation profile extending over a horizontal distance of ~17 km along the Río Tunuyan valley (Figs 1c, 2 and S1 of Supplementary Materials). AHe ages were obtained for 6 samples, in addition to ZHe ages for 4 of these samples. At least 4 and 3 single-grain measurements of apatite and zircon crystals, respectively, were dated for each sample. All AHe analyses were performed at the California Institute of Technology (Pasadena, CA, USA). ZHe dating was conducted at the University of Colorado (Boulder, CO, USA). Details about analytical procedures and sample analysis are reported in the Supplementary Materials. Representing average sample ages with their standard deviation may artificially minimize age dispersions. Therefore, we report in the various figures our AHe and ZHe ages in terms of single grain ages, sample median age and the overall obtained age interval for each sample. All sample locations, obtained grain ages and analytical results are reported in Tables S1 to S3 (Supplementary Materials).

## Thermochronology Results

Obtained AHe ages are similar to - or younger than - the youngest ages of Hoke *et al*.^43^ taken at the same elevations (Fig. 2a). These results appear better resolved than those of Hoke *et al*.^43^ - with a dispersion of single grain ages of at most ~13.8 Ma in the case of the sample collected at 4366 m. Single grain AHe ages range from 5.0 ± 0.4 to 26 ± 0.9 Ma, with median ages of 7.2 to 15.2 Ma increasing with altitude (Fig. 2b). All AHe ages are significantly younger than the crystallization age of the sampled plutons (Fig. 2a). Young and well-resolved ages imply that all samples were located significantly deeper than the PRZ for AHe when exhumation started. Given this, obtained AHe ages can be interpreted as exhumational ages and as indicating the time when each sample crossed the PRZ for AHe. The slight dispersion of AHe ages in the case of some samples is interpreted to reflect minor He retention, probably related to radiation damage of the grains. In this case, the youngest AHe ages of each sample are expected to best reflect exhumational ages (e.g.^52^). As a result, obtained young AHe ages thus reflect Andean continuous exhumation of the Frontal Cordillera from at least ~12–14 Ma (as deduced from the youngest single grain AHe age of our highest sample) to ~4–6 Ma (as deduced from the youngest single grain AHe age of our lowest sample) (Fig. 2b). It also follows that exhumation was already ongoing for some time when the highest sample crossed the PRZ for AHe: the age of this sample therefore provides a minimal estimate for the age of exhumation onset.

Obtained ZHe ages are systematically older than – and therefore consistent with - AHe ages at the same altitude (Fig. 2a). ZHe single grain ages from the 3 highest samples range between 169.1 ± 1.1 and 243.9 ± 2.3 Ma, close to - or slightly younger than - the Permo-Triassic crystallization age of the plutons. The Early Jurassic ZHe ages of some of these samples may reflect minor early basement exhumation related to rifting in the Early Jurassic (e.g.^53^). These observations imply that these high and old samples were likely shallower than the PRZ for ZHe when Andean exhumation initiated in the Cenozoic. In contrast, the lowermost sample yields significantly younger single grain ages, with a large age dispersion ranging from 22.3 ± 0.2 to 115.6 ± 2.1 Ma (Fig. 2a). In particular ZHe single grain ages of this sample correlate with effective Uranium concentration (eU) (Fig. S2 in Supplementary Materials). This indicates that some He may have been retained in the large and high-Uranium content grains, so that the youngest ZHe grain age approximates best (but may still over-estimate) the time at which this sample slowly passed the PRZ for ZHe. Altogether, these observations suggest that the lowest sample was probably located within (or nearby) the PRZ for ZHe when Andean exhumation started. In this case, the youngest ZHe single grain age of this sample could thus be close to the age of exhumation initiation, or at most provide a maximum upper bound on this age.

Conclusively, AHe and ZHe ages indicate that Andean exhumation of the Frontal Cordillera basement clearly initiated before ~12–14 Ma (as derived from the youngest single grain AHe age of 13.0 ± 0.7 Ma of our highest sample), and sometime after ~22 Ma (as derived from the youngest single grain ZHe age of 22.3 ± 0.2 Ma of our lowest sample) (Fig. 2b). More precisely, the hypothesis that the youngest ZHe grain age in fact approximates the age of exhumation initiation is discussed below, by testing its consistency with AHe exhumation ages and published data on nearby syntectonic basins.

## Discussion: Exhumation and Uplift of the Frontal Cordillera Basement at ~33.5°S, and Tectonic Implications

**Figure 3:**
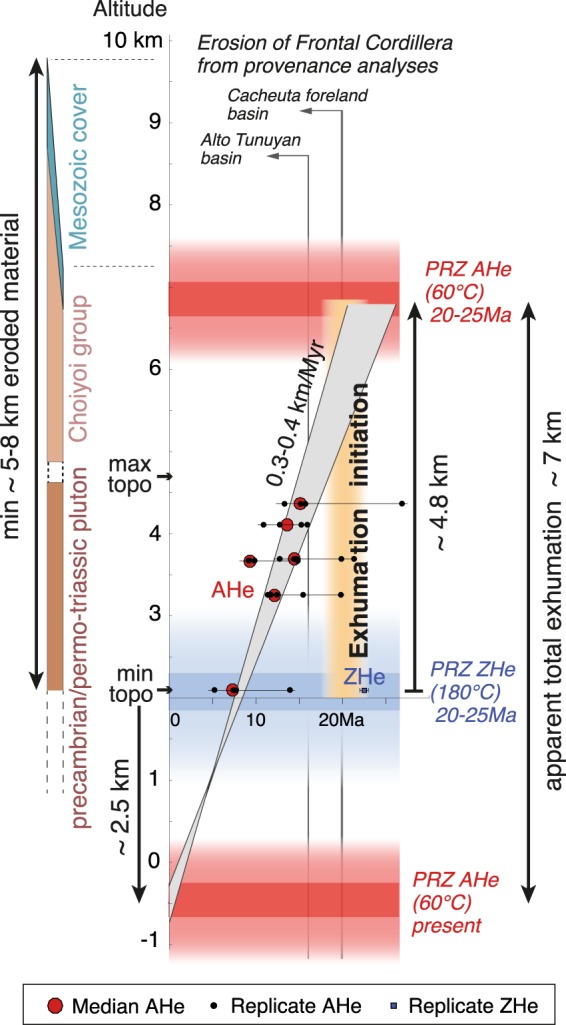
Interpreted apparent thermal and exhumation first-order history of the Frontal Cordillera basement at ~33.5°S based on the AHe and ZHe data provided in this study (center), compared with geological constraints on exhumation (left) and with results on sedimentary provenance analyses of nearby basins^39,40^ (top). See text for further details, and Tables S2 and S3 (Supplementary Materials) for details on the AHe and ZHe ages. PRZ: Partial Retention Zone; AHe and ZHe: apatite and zircon (U-Th)/He ages, respectively. Only average temperatures of ~60 and ~180 °C for the PRZs for AHe and ZHe, respectively, are considered in this first-order reasoning. This results in a simplification of their probable temperature intervals and associated crustal thicknesses, tentatively represented by the red and blue colored bands. Figure was generated with Adobe illustrator CS6 (http://www.adobe.com/fr/products/illustrator.html).

**Figure 4:**
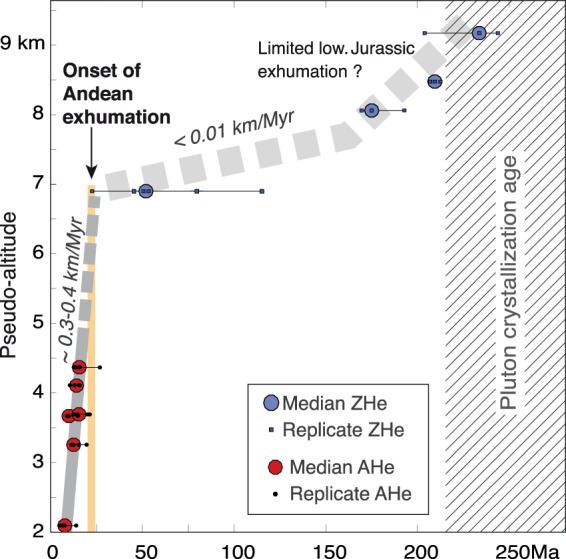
Composite AHe and ZHe age-elevation profile. AHe ages are plotted with their actual altitudes, while ZHe ages are shifted upward by the assumed distance between the PRZs for AHe and ZHe. This distance is determined by considering classical values of 25 °C/km for the upper crustal geothermal gradient and of ~120 °C for the temperature difference between the PRZs for AHe and ZHe. This composite age-elevation plot illustrates a transition from slow crustal cooling to more rapid exhumational cooling by ~20–25 Ma. Figure was generated with Adobe illustrator CS6 (http://www.adobe.com/fr/products/illustrator.html).

The proposed interpretation in terms of age-elevation profile benefits from a significant vertical altitudinal extent (Fig. 2), but suffers from being collected over a quite large horizontal distance (~17 km), as often in this type of studies (Fig. S1 in Supplementary Materials). Over such distances and topographic length-scales, low-temperature isotherms are deflected and only partially follow the topography. The bending of the ~60 °C isotherm is expected to be of ~50% relative to topography, such that obtained AHe ages are closer to each other and actual exhumation rates are consequently ~50% of the obtained apparent rates^49^. On the other hand, the possibility that local relief increased over recent times cannot be discarded, in particular in the glaciated valleys of the Southern Andes. The youngest AHe age is too old to capture exhumation driven by glaciations. However, changes in topographic relief after samples crossed the PRZ for AHe may impact the apparent exhumation rate retrieved at the surface from AHe ages. In this case, the apparent exhumation rate retrieved from AHe ages could be a minimum of the actual rate^48^. These questions cannot be properly addressed without any further appropriate sampling and modeling. This is beyond the scope of this manuscript, and does not have implications on the proposed interpretation in terms of onset of exhumation of the Frontal Cordillera. However, it should be emphasized that only an apparent total exhumation and apparent exhumation rate can be retrieved from the obtained age-elevation profile. The subsequent reasoning will therefore only be based on apparent rates, and will accordingly consider the lowest sample as the reference for altitudes and exhumation (Figs 3–4).

As already noted, the linear AHe age-elevation profile suggests continuous apparent exhumation from at least ~12–14 Ma to ~4–6 Ma (Fig. 2b). From this profile, a minimum apparent exhumation rate of ~0.2 km/Myr is retrieved with a preferred value of 0.3–0.4 km/Myr using a simple fit-by-eye to all AHe age intervals, from at least ~12–14 to ~4–6 Ma (Fig. 2b). The downward extrapolation of the apparent exhumation rate of ~0.3–0.4 km/Myr at depth suggests that a null AHe age is reached ~2.5 km below the modern river level, a depth consistent with the probable modern AHe PRZ when assuming a classical 25 °C/km geotherm (Fig. 3). Furthermore, the large dispersion of ZHe ages of the lowermost sample, with single grains bearing Cenozoic ages significantly younger than the crystallization age of the pluton or than the Early Jurassic ages of some of the highest samples (Figs 2a, 4), indicates that this sample was probably located within or nearby the PRZ for ZHe when exhumation started (Fig. 3). In this case, given the average temperature difference between both PRZs and a ~25 °C/km geotherm, the AHe PRZ at the time of exhumation initiation would be presently ~4.8 km above the lowermost sample (Fig. 3). The upward extrapolation of the apparent exhumation rate suggests that this pre-exhumational AHe PRZ is intercepted at ~20–25 Ma (Fig. 3), a time consistent with the former inference on the maximum age of exhumation initiation of ~22 Ma deduced from the youngest ZHe single grain age. Another way to illustrate this reasoning is to display the AHe and ZHe ages in an age-(pseudo) elevation plot where all data are combined as if pertaining to the same AHe thermochronometric system, i.e. by considering actual altitudes of AHe samples and by shifting the ZHe data upwards by the assumed distance between the PRZs for AHe and ZHe (here 4.8 km for a ~25 °C/km gradient and a ~120 °C temperature difference between both PRZs) (Fig. 4). With this representation, the change from slow crustal cooling to exhumational cooling at an apparent exhumation rate of ~0.3–0.4 km/Myr is clearly illustrated from the break in slope in the age-elevation profile at ~20–25 Ma (Fig. 4). This further supports the idea of exhumation initiation by ~20–25 Ma, ie by the time of ~22 Ma recorded by the youngest ZHe grain (Fig. 4). Based on the AHe age-elevation profile and its extrapolation (Figs 3–4), the newly acquired data are therefore consistent with overall continuous exhumation since its onset. It is acknowledged that the above reasoning using the AHe and ZHe PRZs may seem simplistic as these PRZs are reduced to their average temperatures of ~60 and ~180 °C, respectively (Figs 3–4), and as a classical value for the geotherm is here used for the shallow upper crust. However, it should be viewed as a first-order test to verify the internal consistency of the data and interpretation. The above conclusions meet Hoke *et al*.^43^’s on the probable age for exhumation initiation. They differ however in that the now better-resolved higher apparent exhumation rate does not require a recent (<10 Ma) acceleration of exhumation (Fig. 3).

The results presented here imply that total Cenozoic exhumation since its onset was not sufficient to exhume rocks with fully exhumational ZHe ages, i.e. rocks that were initially deeper than the former ZHe PRZ. This suggests an overall maximum apparent Andean exhumation of ~7 km, consistent with what can be proposed by comparing the probable altitudes of the pre-exhumational and present-day AHe PRZs (Fig. 3). On the other hand, all the samples were collected in Permo-Triassic plutons intruding Precambrian rocks, which were likely initially buried under the ~2–4 km thick Permo-Triassic volcanic series of the Choiyoi group^50^ and possibly under a thin Mesozoic cover (0.5–1 km thick at most, as extrapolated from Riesner *et al*.^23^). Given these geological constraints, the pre-Andean paleo-topography would be at the present-day altitude of at least ~7–10 km, implying a minimum apparent total exhumation of ~5–8 km with respect to the lowest sample (Fig. 3). These independent reasonings, either based on thermochronology or on geology, both confirm an apparent total exhumation of ~7 km. This value is significantly larger that the ≤3 km total exhumation proposed by Hoke *et al*.^43^, even though both the data presented here and Hoke *et al*.^43^’s are overall consistent (Fig. 2a). It cannot be discarded that exhumation is lower where Hoke *et al*.^43^ collected their samples 10–20 km kilometers further southwest along the western flank of the Frontal Cordillera (Fig. 1c). Exhumation may slightly vary laterally within the large-scale basement antiform of the Frontal Cordillera, but significant lateral variations in exhumation are not expected, in particular because reactivation of discrete basement faults during Andean deformation is probably limited^23^. Furthermore, both datasets are expected to record an overall similar cooling history since some of the highest samples of both sets are at a similar structural position within the mountain range (Fig. 1c) and have similar youngest ages (Fig. 2a). Most importantly, the lower estimate of ≤3 km was based on the idea that the complex AHe age pattern of Hoke *et al*.^43^ reflected an exhumed PRZ for AHe, an interpretation refuted by the here better-resolved data (Figs 2, 4). In addition, Hoke *et al*.^43^’s former analysis also relied on interpreting high altitude (~4.5–5 km high) low-relief surfaces observed regionally (Figs 1c, 2) as preserved relicts of a pre-Andean peneplain. However, the nature and origin of these surfaces has not yet been clearly investigated. We note that the Tupungato geological map^54^ reports “Plio-Quaternary alluvial deposits” on top of these surfaces. Recent work in Eastern Tibet suggests that the meaning of similar surfaces may not be straightforward as low-relief landscapes may develop *in situ* by the disruption of the river network during and in response to ongoing tectonic deformation^55^. In any case, the young and better-resolved AHe exhumational ages obtained here beneath these low-relief surfaces indicate that these morphological features are syn- (and not pre-) exhumational. Indeed, the ~500 m separating the highest AHe sample from these morphological features (Fig. 2b) are not sufficient to include the entire pre-Andean AHe PRZ as well as the few km of upper crust above it (Figs 2b, 3).

The data presented here imply that the exhumation of the Frontal Cordillera at ~33.5°S initiated well before ~12–14 Ma and sometime after ~22 Ma (Fig. 2b), with a possible age of onset by ~20–25 Ma from a simple first-order reasoning (Figs 3–4). This is in accordance with the observation that the Frontal Cordillera has been a sedimentary source to the intra-mountainous basins located between the Principal and Frontal Cordilleras since ~16 Ma^39^, as well as for the Cacheuta eastern foreland basin since the first stages of foreland sedimentation at ~20 Ma^40^ (Fig. 3). This indicates that the Frontal Cordillera basement has never been buried under a foreland basin during Andean deformation, a conclusion already reached by Hoke *et al*.^43^ but contrary to classical tectonic interpretations (e.g.^19,21,22,27,30^). As a conclusion, the age of onset of exhumation of the Frontal Cordillera can be refined to ~20 Ma by combining thermochronological data together with sedimentary observations (Fig. 3). The data also indicate that exhumation of the Frontal Cordillera has been overall continuous from at least ~12–14 Ma to ~4–6 Ma (Fig. 2b), and probably since its onset by ~20 Ma (Figs 3–4). The continuity in the apparent basement exhumation over this time period requires that the exhumation of the Frontal Cordillera has been sustained by tectonic uplift rather than been related to the erosion and progressive wearing down of a pre-existing topographic high. The data presented here on the exhumation of the Frontal Cordillera are therefore interpreted as reflecting its early Andean uplift starting by ~20 Ma at ~33.5°S - or most probably slightly before ~20 Ma in case of a delay in the erosional response following the onset of uplift.

**Figure 5:**
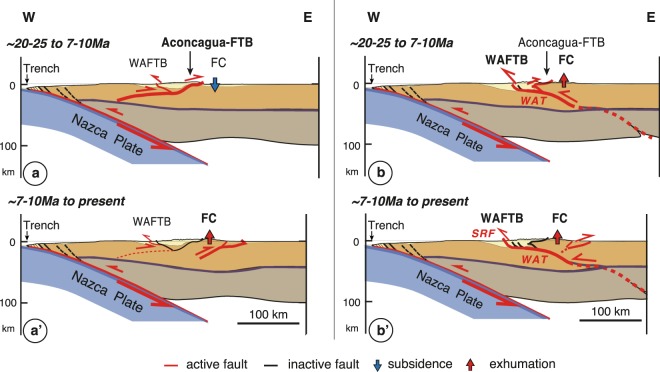
Schematic tectonic evolution of the Andes at ~33.5°S (cross-section located on Fig. 1b) and associated uplift and exhumation of the Frontal Cordillera, according to two conceptual models of this mountain range at this latitude. Moho geometry derived from published receiver functions^58^. (**left**) Tectonic evolution of the Andes considering that deformation initiated on the east-vergent Aconcagua FTB (**a**) and then propagated east of the Frontal Cordillera (a’) in an eastward sequential propagation of deformation (e.g.^19–22,27,30^). In this case uplift and exhumation of the Frontal Cordillera would be sustained by an east-vergent crustal-scale ramp, but would initiate by ~7–10 Ma, ie much later than documented by thermochronology (^43^; this study) and provenance studies of nearby sedimentary basins^39,40^. (**right**) Tectonic evolution of the Andes considering that deformation is primarily sustained along a west-vergent crustal-scale basal detachment termed the West Andean Thrust (WAT)^23,24^. Deformation on the WAT has been proposed to initiate by ~20–25 Ma (**b**) (e.g.^23^), in accordance with the here documented onset of exhumation of the Frontal Cordillera at ~20 Ma. Uplift and exhumation of the Frontal Cordillera would have been sustained to present-day by a crustal-scale ramp of the WAT that transfers slip westward to the West Andean FTB along the western front of the Andes (**b**-b’). SRF: San Ramón Fault; WAT: West Andean Thrust; Aconcagua FTB: Aconcagua fold-and-thrust belt; WAFTB: West Andean fold-and-thrust belt; FC: Frontal Cordillera. Figure was generated with Adobe illustrator CS6 (http://www.adobe.com/fr/products/illustrator.html).

Given the large scale of the Frontal Cordillera basement culmination at ~33.5°S (Fig. 1c), its exhumation and uplift needs to be sustained by slip on a crustal-scale thrust ramp at depth. Presently, such structure is proposed by various structural cross-sections of the Andes at this latitude. These structural interpretations can be simplified into two main conceptual views (Fig. 5). On one side, most models propose two main east-vergent crustal-scale ramps, one connected to the emblematic Aconcagua FTB, the other at depth beneath the Frontal Cordillera (Fig. 5a-a’). It is suggested that deformation initiated on the east-vergent Aconcagua FTB (Fig. 5a). There is geological evidence that this fold-and-thrust belt has been active from at least ~17–18 Ma to ~9 Ma (e.g.^23^ and references therein). It is proposed that deformation subsequently propagated east and beneath the Frontal Cordillera by ~7–10 Ma, in a sequential eastward propagation of deformation (e.g.^19–22,27,30^) (Fig. 5a’). These models imply that during the progressive formation of the Aconcagua FTB, the Frontal Cordillera basement was in the undeformed footwall or foreland of this fold-and-thrust belt, thus underthrust, downflexed and eventually buried beneath an eastern foreland basin. This is in clear contradiction with the ongoing exhumation of the Frontal Cordillera at the same time as documented by thermochronological data (^43^; this study), as well as by that on sedimentary provenances of nearby basins^39,40^. Alternatively, a latest structural model proposes a west-vergent crustal-scale ramp beneath the Frontal Cordillera that connects at shallow depth with the West Andean FTB along the western flank of the mountain belt^23,24^ (Fig. 5b-b’). Such basement structure beneath the orogen is termed the West Andean Thrust (WAT). This model suggests that protracted westward motion on the WAT has sustained continuous uplift of the Frontal Cordillera, coeval with the westward propagating deformation within the West Andean FTB^23,24^ (Fig. 5b-b’). Deformation within the West Andean FTB is documented to have initiated by ~20–25 Ma (Fig. 5b) and to be still active^34^, as illustrated by the presently active San Ramón Fault along the mountain front east of Santiago de Chile^35^ (Fig. 5b’). In this model, the concomitant Aconcagua FTB is interpreted as a secondary shallow roof structure, passively back-thrusted over the uplifting Frontal Cordillera basement^23^ (Fig. 5b). This recent structural model of the Andes at ~33–33.5°S therefore implies that uplift of the Frontal Cordillera initiated by ~20–25 Ma, concomitant to the initiation of deformation across the West Andean FTB, and that it has since been sustained. These model inferences on the uplift of the Frontal Cordillera sustained since ~20–25 Ma are compatible with the early onset of continuous exhumation of this basement high since ~20 Ma, as interpreted from our thermochronological data in combination with constraints on the provenance of sediments in nearby basins (Fig. 5b-b’).

## Conclusion

In this study, new low-temperature thermochronological data are provided along an elevation profile within the core of the Frontal Cordillera basement at ~33.5°S. Total apparent exhumation has remained limited to ~7 km, allowing for keeping a record of the probable earlier stages of exhumation in the ZHe thermochronometric system. It is shown that the exhumation of the Frontal Cordillera basement initiated well before ~12–14 Ma and sometime after ~22 Ma, and most probably by ~20 Ma when thermochronological constraints are combined with data on detrital sediments in nearby basins^39,40^. Because the apparent exhumation of this large-scale basement antiform appears overall continuous over time, it is proposed that it has been sustained by tectonic uplift related to slip on a crustal-scale ramp. Existing conceptual models of the Andes at ~33–33.5°S are discussed, and it is found that only the most recent models implying an initial westward basement vergence of the Andes at these latitudes^23,24^ are consistent with the data on the exhumation of the Frontal Cordillera. At a larger scale, an overall westward basement vergence of the Andes at ~33.5°S would also fit to the first-order the westward trend towards older low-temperature thermochronological ages documented within the Principal Cordillera further west^56^. Future modeling of the thermochronologic data is therefore needed to further quantitatively explore the existing structural models of the Southern Central Andes (~33.5°S).

## Supplementary information

Supplementary Materials

Table S1

Table S2

Table S3

## Data Availability

All data used in this study are available from the supplementary information files (location of samples and details on (U-Th)/He analyses in Tables S1 to S3) and from cited references ((U-Th)/He ages from Hoke *et al*.^43^).
