# Supplementary material for: Early exhumation of the Frontal Cordillera (Southern Central Andes) and implications for Andean mountain-building at ~33.5°S
Source: Sci Rep. 2019 May 28;9:7972. doi: 10.1038/s41598-019-44320-1 (PMC6746751; doi:10.1038/s41598-019-44320-1)
Supplement: Supplementary file 1 — Supplementary Materials [file 41598_2019_44320_MOESM1_ESM.docx]

Scientific Reports

Supporting information for

**Early exhumation of the Frontal Cordillera (Southern Central Andes) and implications for Andean mountain-building at ~33.5°S.**

Magali Riesner (1,2), Martine Simoes (1), Robin Lacassin (1) and Daniel Carrizo (3).

1. Université de Paris, Institut de physique du globe de Paris, CNRS, F-75005 Paris, France ; (2) now at CEA, DAM, DIF, F-91297 Arpajon, France; (3) Advanced Mining Technology Center, Univ. de Chile, Santiago, Chile.

Corresponding author: Magali Riesner ([magali.riesner@gmail.com)](mailto:email@address.edu))

**Introduction**

In this supplementary information, we first discuss the observations of Darwin (1845, 1846) pointing out the existence of conglomeratic clasts from the Frontal Cordillera early within the intramountainous basins of the Andes at ~33.5°S. In particular we aim here at correcting for some wrong quotations of this pioneer work.

Then, we provide further details on our sampling site (Figure S1 and Table S1). We also provide tables for the apatite (Table S2) and zircon (Table S3) (U-Th)/He ages obtained at the California Institute of Technology (Pasadena, CA, USA) and at the University of Colorado (Boulder, CO, USA), respectively. At least 4 and 3 single-grain measurements of apatite and zircon crystals, respectively, were dated for each sample. All single grain ages are reported. From these single grain ages, a median age and an age interval are determined for each sample. Median ages are calculated from the median of all single grain ages for the corresponding sample. Age intervals cover a time span from the minimum possible age (youngest single grain age - uncertainty on this age) to the maximum possible age (oldest single grain age + uncertainty on this age) for each sample. This age range is represented by horizontal lines on Figures 2 to 4. We also provide details about dating procedures. Figure S2 illustrates the correlation between effective Uranium content and (U-Th)/He age for the zircons of the lowest sample.

**The Frontal Cordillera as an early sediment source for the intramountainous basins of the Andes at ~33.5°S: a discussion on the early observations by Darwin.**

Seminal work by C. Darwin ^1,2^ resulting from his two traverses of the Andes, at the latitude of Santiago de Chile and of Mendoza, is often referred as the first modern geological description of the Andean orogeny. In this respect, a recurrent remark is that “ Based on the study of the clast provenance of the synorogenic deposits, Darwin was able to recognize the early uplift of the Cordillera Principal, followed by that of the Cordillera Frontal”. Same type of claim is found in recent work by Giambiagi et al ^3^: “It is interesting to remark how Darwin, based on a provenance analysis of conglomeratic clasts, was able to infer the order and the relative uplift age of each mountain chain across this section of the Andes.” After carefully reading C. Darwin detailed reports ^1,2^, we understand that these above statements are misquoting and misinterpreting Darwin’s original text. As an evidence for this, we hereafter paste Darwin’s description of the clasts from the Alto Tunuyan basin as printed in his “Geological Observations on South America” book ^2^:

*“ The Portillo or Eastern Chain. — The valley of Tenuyan, separating the Peuquenes and Portillo lines, is, as estimated by Dr. Gillies and myself, about twenty miles in width; the lowest part, where the road crosses the river, being 7,500 feet above the sea-level. The pass on the Portillo line is 14,365 feet high (1100 feet higher than that on the Peuquenes), and the neighbouring pinnacles must, I conceive, rise to nearly 16,000 feet above the sea. The river draining the intermediate valley of Tenuyan, passes through the Portillo line. To return to our section;—shortly after leaving the lower beds [P2] of the gypseous formation, we come to grand masses of a coarse, red conglomerate [V], totally unlike any strata hitherto seen in the Cordillera. This conglomerate is distinctly stratified, some of the beds being well defined by the greater size of the pebbles: the cement is calcareous and sometimes crystalline, though the mass shows no signs of having been metamorphosed. The included pebbles are either perfectly or only partially rounded: they consist of purplish sandstones, of various porphyries, of brownish limestone, of black calcareous, compact shale precisely like that in situ in the Peuquenes range, and containing some of the same fossil shells; also very many pebbles of quartz, some of micaceous schist, and numerous, broken, rounded crystals of a reddish orthitic or potash feldspar (as determined by Professor Miller), and these from their size must have been derived from a coarse-grained rock, probably granite. From this feldspar being orthitic, and even from its external appearance, I venture positively to affirm that it has not been derived from the rocks of the western ranges; but on the other hand it may well have come, together with the quartz and metamorphic schists, from the eastern or Portillo line, for this line mainly consists of coarse orthitic granite. The pebbles of the fossiliferous slate and of the purple sandstone, certainly have been derived from the Peuquenes or western ranges.”* (Darwin 1846, p. 182)

Note that the "Peuquenes range" and the " eastern Portillo line" correspond to the Principal Cordillera (sedimentary rocks of Mesozoic to Cenozoic age) and to the Frontal Cordillera (older metamorphic and granitic rocks), respectively. A comparable description is found in C. Darwin (1845)'s journal ^1^ from which we quote the following sentences:

*“I was astonished to find that this conglomerate was partly composed of pebbles, derived from the rocks, with their fossil shells, of the Peuquenes range; and partly of red potashgranite, like that of the Portillo. Hence we must conclude, that both the Peuquenes and Portillo ranges were partially upheaved and exposed to wear and tear, when the conglomerate was forming…”* (Darwin 1845, p. 320)

Darwin thus describes the Alto Tunuyan conglomerates bearing clasts from both the western Meso-Cenozoic ranges of the Principal Cordillera and from the eastern basement of the Frontal Cordillera, which implies coeval erosion and exhumation of the two structural units.

Then he builds on a discussion upon some prejudicial ideas typical of the 19th century. Darwin ^1,2^ considers that the way crystalline mountain ranges reach high elevations and deform their surrounding rocks is by plutonic (“injection”) processes in the recent past, and therefore from this a priori argues for a very recent uplift of the Portillo granitic range (i.e. Frontal Cordillera). To illustrate this reasoning we copy below an extract of his “Concluding remarks on the Portillo range” ^2^:

*“The red orthitic granite now forms, as we have seen, the main part of the Portillo chain: it is injected in dikes not only into the mica-schist and white granites, but into the laminated sandstone, which it has metamorphosed, and which it has thrown off, together with the conformably overlying coloured beds and stratified conglomerate, at an angle of forty-five degrees. To have thrown off so vast a pile of strata at this angle, is a proof that the main part of the red granite (whether or not portions, as perhaps is probable, previously existed) was injected in a liquefied state after the accumulation both of the laminated sandstone and of the conglomerate.”* (Darwin 1846, p.186)

We thus conclude that Darwin's speculation of a recent uplift of the Frontal Cordillera does not come from his observations of the synorogenic clast provenance - which rather imply a coeval exhumation of both Principal and Frontal Cordillera -, but from an a 19th century *a priori* on the way metamorphic mountain ranges grow. We note the apparently conflicting observation that the Alto Tunuyan conglomerates bear granitic clasts from the Frontal Cordillera but has been deformed (“thrown off”) by its rise “in a liquefied state.” These observations are in fact clearly compatible with a protracted uplift and exhumation of the Frontal Cordillera during and after deposition of the conglomerates (given that we abandon the idea of a recent “injection” of the granitic rocks, which are now known to be of late Paleozoic age).

**Some information on how to access Darwin's work**

Darwin, C. R. 1845. *Journal of researches into the natural history and geology of the countries visited during the voyage of H.M.S. Beagle round the world, under the Command of Capt. Fitz Roy, R.N.* 2d edition. London: John Murray.

<http://darwin-online.org.uk/content/frameset?pageseq=333&itemID=F14&viewtype=side>

Darwin, C. R. 1846. Geological observations on South America. Being the third part of the geology of the voyage of the Beagle, under the command of Capt. Fitzroy, R.N. during the years 1832 to 1836. London: Smith Elder and Co.

<http://darwin-online.org.uk/content/frameset?pageseq=196&itemID=F273&viewtype=side>

**Sample locations and analytical data.**


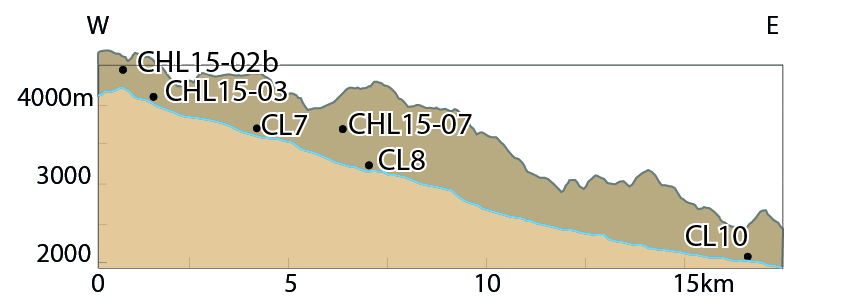


**Figure S1**: Position of dated samples within a topographic 4 km wide swath profile of our sampling site. Blue line indicates Tunuyan river level. Topography is extracted from ASTER digital elevation model (~30 m resolution).


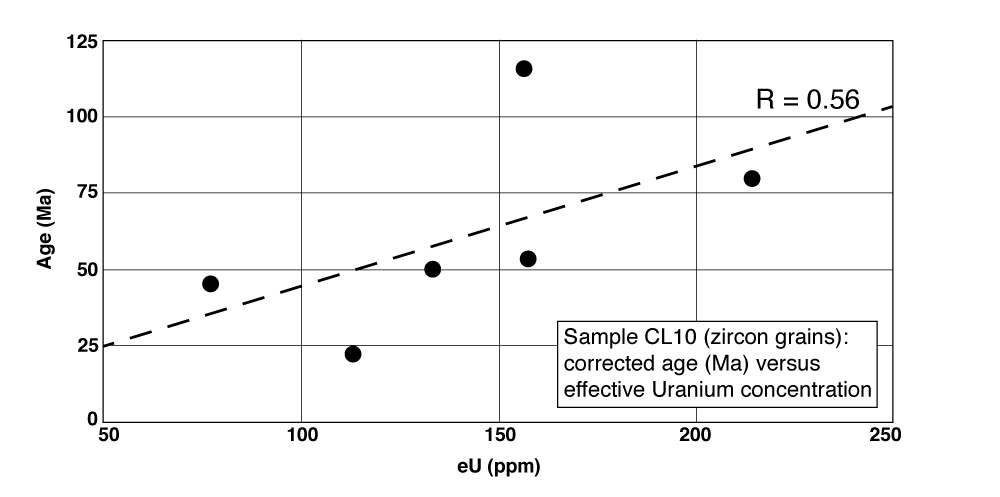


**Figure S2**. eU vs ZHe age for the particular case of the youngest single grain age of our lowest sample (sample CL10).

**Table S1**: Location of collected samples and list of completed analyses. Column headings:

**Lat (°S)** and **Lon (°W)**: location of sampling site in terms of latitude and longitude (WGS84)

**Elevation (m)** : elevation of sample in m

**AHe** and **ZHe**: crossed when (U-Th)/He analyses were performed on apatite or zircon, respectively

**Table S2**: (U-Th)/He apatite age results and analytical data. Column headings :

**Sample Name**: Sample Name

**grain**: separate grain analyses from the same sample

**Lat (°S)** and **Lon (°W)**: location of sampling site in terms of latitude and longitude (WGS84)

**Elevation (m)** : elevation of sample in m

**U (ppm)**: the amount of total U in the sample, measured via isotope dilution on an ICP-MS. The following **1σ ppm** column is 1-sigma analytical uncertainty.

**Th (ppm)**: the amount of total Th in the sample, measured via isotope dilution on an ICP-MS. The following **1σ ppm** is 1-sigma analytical uncertainty.

**He (nmol/g)**, the amount of He measured in the crystal via isotope dilution. This includes all of the He if multiple degassing steps were required.

**Sm (ppm)**, the amount of total Sm in the sample, measured via isotope dilution on an ICP-MS.

**mass (μg)**: mass (in micrograms) calculated based solely on the volume of the crystal (determined from the measurements) and average apatite density.

**Ft**: He alpha ejection correction calculated using the method of Ketcham et al ^4^. Ft is a measure of the amount of He that was ejected from the crystal. A Ft of 1.00 would indicate that no He has been lost. This is a purely geometric correction.

**r (μm)** and **l (μm)**: measurements of the grain dimensions (radius and length, respectively) in micrometers. Each grain is measured from two different angles to ensure that the grain geometry is fully captured. These values are used to calculate alpha ejection corrections and crystal volumes and masses using the techniques described by Ketcham et al. ^4^

**Raw Age (Ma)**, the age calculated directly from He, U, Th, and Sm measurements. This age, as well as the corrected age, are calculated iteratively using equation #34 from Ketcham et al. ^4^.

**Dim Mass (μg)**, The dimensional mass (in micrograms) is calculated based solely on the volume of the crystal (determined from the measurements) and average apatite density.

**Corrected age (Ma)**: The alpha-ejection corrected age, essentially equal to the Raw Age divided by the alpha-ejection correction, calculated iteratively using equation #34 from Ketcham et al. ^4^. The following **1σ (Ma)** column is 1-sigma uncertainty.

**Median age (Ma)** : the median age calculated from each single grain age for each selected aliquot. The **Youngest age (Ma)** is calculated by taking the oldest single grain age and subtracting from it its uncertainty. The **Oldest age (Ma)** is calculated by taking the oldest single grain age and adding to it its uncertainty.

**Durango test** (bottom lines in the table): analytical results for variable tests on Durango apatites, used for calibration.

**Table S3**: (U-Th)/He zircon age results and analytical data. Column headings :

**Sample Name**: Sample Name (in bold), the subscripts such as _z01 refer to separate grain analyses from the same sample.

**Lat (°S)** and **Lon (°W)**: location of sampling site in terms of latitude and longitude (WGS84).

**Elevation (m)** : elevation of sample in m.

**length (μm)** and **width (μm)**: measurements of the grain dimensions in micrometers. Each grain is measured from two different angles (1 and 2) to ensure that the grain geometry is fully captured. These values are used to calculate alpha ejection corrections and crystal volumes and masses using the techniques described by Ketcham et al. ^4^.

**2X Term**: Notes whether (Y) or not (N) the grain is doubly terminated.

**Np**: The number of pyramidal terminations of the grain, used in the alpha ejection correction.

**Dim Mass (μg)**: The dimensional mass (in micrograms) is calculated based solely on the volume of the crystal (determined from the measurements) and average zircon density.

**rs (μm)**: The radius of a sphere with an equivalent surface area to volume ratio as your crystal. This value is required for thermal modeling of the grain ages using HeFTy ^5^.

**4He (nmol/g)**, the amount of 4He measured in the crystal via isotope dilution. This includes all of the He if multiple degassing steps were required. The **1σ (nmol/g)** column is 1-sigma analytical uncertainty.

**U (ppm)**: the amount of total U in the sample, measured via isotope dilution on an ICP-MS. The following **1σ (ppm)** column is 1-sigma analytical uncertainty.

**Th (ppm)**, the amount of total Th in the sample, measured via isotope dilution on an ICP-MS. The following **1σ (ppm)** column is 1-sigma analytical uncertainty.

**Sm (ppm)**, the amount of total Sm in the sample, measured via isotope dilution on an ICP-MS. The following **1σ (ppm)** column is 1-sigma analytical uncertainty. A value of 0.00 indicates measurements that are not larger than analytical uncertainty.

**eU (ppm):** the effective Uranium concentration, a measurement of the total amount of radiation experienced by the crystal, equivalent to U + .235Th.

**4He (ncc)**: the total amount of He, blank corrected, in nano-cc’s, measured from the sample. This is not referenced to the grain mass. The following **1σ (ncc)** column is 1-sigma analytical uncertainty.

**Re (%)**: the percent of the total He that was degassed during the first laser extraction. For apatites, this number should be 99.9% or higher. Lower values are thought to indicate the presence of inclusions or other low-diffusivity zones. For zircons this value is not as instructive.

**U (ng)**: the total amount of Uranium measured in the sample in nanograms. **Th (ng)**: the total amount of Thorium measured in the sample in nanograms. **Sm (ng)** the total amount of Samarium measured in the sample in nanograms. This is not referenced to grain mass. The **1σ (ng)** columns are 1-sigma analytical uncertainties.

**Th/U**: The Thorium/Uranium ratio for the sample.

**Raw Age (Ma)**: the age calculated directly from He, U, Th, and Sm measurements. The following **1σ (Ma)** column is 1-sigma analytical uncertainty. This date, as well as the corrected age, are calculated iteratively using equation #34 from Ketcham et al. ^4^.

**Ft**: The alpha ejection correction calculated using the method of Ketcham et al. ^4^. Ft is a measure of the amount of He that was ejected from the crystal. An Ft of 1.00 would indicate that no He had been lost.

**Corrected Age (Ma)**: The alpha-ejection corrected age, essentially equal to the Raw Age divided by the alpha-ejection correction, calculated iteratively using equation #34 from Ketcham et al. ^4^.

**1σ Unc (Ma)**, Uncertainty in millions of years not including the estimate for the uncertainty in the alpha ejection correction, and only including direct analytical uncertainties.

**Median age (Ma)** : the median age calculated from each single grain age for each

selected aliquot. The **Youngest age (Ma)** is calculated by taking the oldest single grain age and subtracting from it its uncertainty. The **Oldest age (Ma)** is calculated by taking the oldest single grain age and adding to it its uncertainty.

Bottom line in the table: results for **Fish Canyon zircons** run in conjunction, used for calibration.

**Analytical procedures for apatite (U-Th)/He dating.**

Apatites have been analyzed following standard procedures used at the California Institute of Technology (U-Th)/He laboratory ^6,7^. These are reported in detail in Sousa et al ^5^.

**Analytical procedures for zircon (U-Th)/He dating**

Analyses carried out at the University of Colorado Boulder TRaIL (Thermochronology Research and Instrumentation Lab) used the following methods. Individual mineral grains are handpicked using a Leica M165 binocular microscope equipped with a calibrated digital camera and capable of both reflected and transmitted, polarized light. The grains are screened for quality, including crystal size, shape, and the presence of inclusions. After characterization, grains are placed into small Nb tubes that are then crimped on both ends. This Nb packet is then loaded into an ASI Alphachron He extraction and measurement line. The packet is placed in the UHV extraction line (~3 X 10-8 torr) and heated with a 25W diode laser to ~800-1100°C for 5 to 10 minutes to extract the radiogenic 4He. The degassed 4He is then spiked with approximately 13 ncc of pure 3He, cleaned via interaction with two SAES getters, and analyzed on a Balzers PrismaPlus QME 220 quadrupole mass spectrometer. This procedure is repeated at least once to ensure complete mineral degassing. Degassed grains are then removed from the line, and taken to a Class 10 clean lab for dissolution.

Zircons are dissolved using Parr large-capacity dissolution vessels in a multi-step acid-vapor dissolution process. Grains (including the Nb tube) are placed in Ludwig-style Savillex vials, spiked with a 235U - 230Th - 145Nd tracer, and mixed with 200 μl of Optima grade HF. The vials are then capped, stacked in a 125 mL Teflon liner, placed in a Parr dissolution vessel, and baked at 220°C for 72 hours. After cooling, the vials are uncapped and dried down on a 90°C hot plate until dry. The vials then undergo a second round of acid-vapor dissolution, this time with 200 μl of 6N Optima grade HCl in each vial that is baked at 200°C for 24 hours. Vials are then dried down a second time on a hot plate. Once dry, 200 μl of a 7:1 HNO3:HF mixture is added to each vial, the vial is capped, and cooked on the hot plate at 90°C for 4 hours. Once the minerals are dissolved, they are diluted with 1 to 3 mL of doubly-deionized water, and taken to the ICP-MS lab for analysis. Sample solutions, along with normal solutions and blanks, are analyzed for U, Th, and Sm content using either a Thermo Element 2 magnetic sector ICP-MS or an Agilent 7900 quadrupole ICP-MS. After the U, Th, and Sm contents are measured, He dates and all associated data are calculated on a custom spreadsheet using the methods described in Ketcham et al. ^4^. Every batch of samples includes standards run sporadically throughout the process to monitor procedures and maintain consistency from run to run. Long term averages of Fish Canyon Tuff zircons run in the CU TRaIL are 28.7 ± 1.8 Ma (n=150) and 31.1 ± 2.1 (n=85), respectively. The specific value obtained during the analyses here is reported at the bottom of Table S3.

**References**

1 Darwin, C. R. *Journal of researches into the natural history and geology of the countries visited during the voyage of H.M.S. Beagle round the world, under the Command of Capt. Fitz Roy, R. N.* 2nd edition edn, (John Murray, 1845).

2 Darwin, C. R. *Geological observations on South America. Being the third part of the geology of the voyage of the Beagle, under the command of Capt. Fitzroy, R.N. during the years 1832 to 1836.*, (Smith Elder and Co., 1846).

3 Giambiagi, L., Tunik, M., Ramos, V. & Godoy, E. The High Andean Cordillera of central Argentina and Chile along the Piuquenes Pass-Cordon del Portillo transect: Darwin's pioneering observations compared with modern geology. *Revista de la Asociación Geológica Argentina* **64**, 43-49 (2009).

4 Ketcham, R. A., Gautheron, C. & Tassan-Got, L. Accounting for long alpha-particle stopping distances in (U-Th-Sm)/He geochronology: Refinement of the baseline case. *Geochemica and Cosmochemica Acta* **75**, 7779-7791, doi:10.1016/j.gca.2011.10.011 (2011).

5 Ketcham, R. A. Forward and inverse modeling of low temperature thermochronometry data. *Reviews in Mineralogy and Geochemistry* **58**, 275-314 (2005).

6 Farley, K. A. (U-Th)/He dating: Techniques, calibrations, and applications. *Reviews in Mineralogy and Geochemistry* **47**, 819–844. (2002).

7 Farley, K. A., Wolf, R. A. & Silver, L. T. The effects of long alpha-stopping distances on (U–Th)/He ages. *Geochemica and Cosmochemica Acta* **60**, 4223-4229 (1996).
